# Molecular Events in Immune Responses to Sublingual Influenza Vaccine with Hemagglutinin Antigen and Poly(I:C) Adjuvant in Nonhuman Primates, Cynomolgus Macaques

**DOI:** 10.3390/vaccines12060643

**Published:** 2024-06-08

**Authors:** Tetsuro Yamamoto, Makoto Hirano, Fusako Mitsunaga, Kunihiko Wasaki, Atsushi Kotani, Kazuki Tajima, Shin Nakamura

**Affiliations:** 1 Innovation Research Center, EPS Holdings, Inc., 2-1 Tsukudohachimancho, Shinjuku-ku, Tokyo 162-0815, Japan; yamamoto.tetsuro061@eps.co.jp (T.Y.); wasaki.kunihiko377@eps.co.jp (K.W.); kotani.atsushi332@eps.co.jp (A.K.); tajima.kazuki374@eps.co.jp (K.T.); 2 EP Mediate Co., Ltd., 1-8 Tsukudocho, Shinjuku-ku, Tokyo 162-0821, Japan; 3 Research Center, EPS Innovative Medicine Co., Ltd., 1-8 Tsukudocho, Shinjuku-ku, Tokyo 162-0821, Japan; 4 Intelligence & Technology Lab, Inc., 52-1 Fukue, Kaizu-cho, Kaizu 503-0628, Japan; mhirano@itechlab.co.jp (M.H.); mitsunaga@itechlab.co.jp (F.M.); 5 Biomedical Institute, NPO Primate Agora, 52-2 Fukue, Kaizu-cho, Kaizu 503-0628, Japan

**Keywords:** monkey, prophylaxis, efficacy, tolerance, *CLEC4G*, LSECtin

## Abstract

Sublingual vaccines offer the benefits of inducing mucosal immunity to protect against respiratory viruses, including Severe Acute Respiratory Syndrome Coronavirus 2 (SARS-CoV-2) and influenza, while also enabling needle-free self-administration. In a previous study, a sublingual SARS-CoV-2 vaccination was created by combining a recombinafigureCoV-2 spike protein receptor-binding domain antigen with a double strand RNA Poly(I:C) adjuvant. This vaccine was tested on nonhuman primates, Cynomolgus macaques. This study examined the immune and inflammatory responses elicited by the sublingual influenza vaccine containing hemagglutinin (HA) antigen and Poly(I:C) adjuvants, and assessed the safety of this vaccine in nonhuman primates. The Poly(I:C)-adjuvanted sublingual vaccine induced both mucosal and systemic immunities. Specifically, the sublingual vaccine produced HA-specific secretory IgA antibodies in saliva and nasal washings, and HA-specific IgA and IgG were detected in the blood. This vaccine appeared to be safe, as judged from the results of blood tests and plasma C-reactive protein levels. Notably, sublingual vaccination neither increased the production of inflammation-associated cytokines—IFN-alpha, IFN-gamma, and IL-17—in the blood, nor upregulated the gene expression of proinflammatory cytokines—IL12A, IL12B, IFNA1, IFNB1, CD69, and granzyme B—in white blood cells. Moreover, DNA microarray analyses revealed that sublingual vaccination evoked both enhancing and suppressing expression changes in genes associated with immune-related responses in cynomolgus monkeys. Therefore, the sublingual vaccine with the Poly(I:C) adjuvant is safe, and creates a balanced state of enhancing and suppressing the immune-related response.

## 1. Introduction

Influenza is a highly contagious respiratory disease caused by influenza A and B viruses. Symptoms are usually cold-like, but some patients develop pneumonia, requiring hospitalization. The World Health Organization estimates that influenza epidemics cause 3–5 million cases of severe illness and 290,000–650,000 deaths annually worldwide [1]. The influenza virus, a single-stranded RNA virus, is prone to genetic mutation and can antigenically “drift,” leading to seasonal epidemics every winter. Seasonal influenza is considered to be a vaccine-preventable disease, but the current mainstay of needle-and-syringe vaccines has shortcomings, such as relative ineffectiveness in elderly people [2,3].

Optimizing administration methods is a key strategy to enhance the effectiveness of influenza vaccines. The influenza virus enters the oral or nasal cavities and establishes infection in the upper respiratory tract mucosae. Therefore, these mucosae serve as the first line of defense against pathogens such as the influenza virus and Severe Acute Respiratory Syndrome Coronavirus 2 (SARS-CoV-2). Mucosal protection generally operates through antibody-mediated and cytotoxic T-cell responses, which can be triggered to some extent by needle-and-syringe vaccines.

Sublingual vaccinations, similar to nasal vaccines, primarily stimulate mucosal immune responses in both the upper and lower respiratory tract, as well as a systemic response [4]. Sublingual vaccinations have the benefit of being safer than nasal vaccines, as the latter may have negative effects on the brain, central nervous system, or lungs [5,6]. Moreover, the administration of sublingual vaccines eliminates the need for needles, improving patient compliance and enabling self-administration without the assistance of medical personnel.

However, the sublingual method of administering vaccines faces practical challenges. For example, there is a mucin barrier that prevents the vaccine from reaching immune cells, and there is a lot of saliva, which lowers the concentration of the vaccine. We addressed these issues in a previous study using cynomolgus macaques by applying N-acetyl cysteine (NAC), a mild reducing agent that breaks down the mucin layer, to the underside of the tongue [7]. Furthermore, we reduced the dilution of saliva by providing an anesthetic, namely a mixture of medetomidine and ketamine, with the aim of decreasing saliva production during vaccination [7]. Later on, we provided evidence to support the safety and effectiveness of a sublingual SARS-CoV-2 vaccination that includes the receptor binding domain (RBD) of the spike (S) glycoprotein of SARS-CoV-2 [8].

Nowadays, adjuvants are integral components of medical and veterinary vaccines, excluding live-attenuated and recombinant adenovirus-vectored vaccines. The primary role of adjuvants is to stimulate innate immune responses and elicit antigen-specific adaptive immune responses [9]. Adjuvants can be broadly categorized into delivery systems and immunostimulants. Delivery systems consist of carrier materials that load antigens, enhancing their uptake and presentation by antigen-presenting cells (APCs), thereby facilitating antigen presentation. Conversely, immunostimulants are pathogen-associated molecular pattern (PAMP) molecules that induce the maturation and activation of APCs by targeting Toll-like receptors and other pattern recognition receptors [10].

Examples of delivery system adjuvants include MF59 and AS03, which are both oil-in-water nanoemulsions. These adjuvants have received approval for use in influenza vaccines [11]. Conversely, double-stranded polyinosinic:polycytidylic acid (Poly(I:C)), a TLR3 agonist, falls under the category of immunostimulant adjuvants. However, Poly(I:C) has not yet received approval due to its side effects, including fever and proinflammatory cytokine production, when administered intramuscularly. In a previous study, we performed a direct comparison between AS03 and Poly(I:C) adjuvants in the sublingual vaccination of cynomolgus macaques and found that Poly(I:C) exhibited superior safety profiles compared to AS03 [8].

As mentioned above and in previous papers [7,8], we demonstrated the safety and efficacy of poly(I:C)-adjuvanted SARS-CoV-2 vaccines administered via the sublingual route in non-human primates. Poly(I:C) adjuvant appeared to be safe, even though its adverse side effects were reported in cases of nasal administration in mice [6]. The side effects of Poly(I:C) adjuvant were overestimated due to nasal administration in mice, because there are marked differences in immune systems between primates and rodents. We also reported the use of NAC to break down the mucin barrier, which inhibits the vaccine from reaching sublingual immune cells [7]. Moreover, our previous works uncovered previously unknown molecular events in an immune response mediated by the sublingual vaccine [8]. These insights offered valuable information about the progress in the development of sublingual vaccines for respiratory viruses. Nevertheless, further investigation is necessary.

In this study, in continuation of the previous studies on sublingual SARS-CoV-2 vaccines [7,8], we aimed to assess the safety and efficacy of a sublingual Poly(I:C)-adjuvanted vaccine comprising a different respiratory virus antigen. We examined sublingual influenza vaccines formulated with influenza hemagglutinin (HA) antigen and Poly(I:C) adjuvant in non-human primates. Furthermore, DNA microarray analysis was performed to elucidate molecular mechanisms underlying immune-related responses induced by the sublingual vaccine

## 2. Materials and Methods

### 2.1. Reagents, Wares, and Antibodies

The following materials were used in this study: phosphate-buffered saline (PBS) (Nissui, Tokyo, Japan), polyester swabs (JCB, Industry Limited, Tokyo, Japan), filter spin columns (Norgen Biotek Corp., Thorold, ON, Canada), Nunc-immuno module, F8 Maxisorp (Thermo Fisher Scientific K.K., Yokohama, Japan), streptavidin-HRP Conjugate (SA-HRP) (Invitrogen-Thermo Fisher Scientific K.K., Gifu, Japan), and tetramethyl benzidine (TMB) (Sigma-Aldrich Co. LLC, Tokyo, Japan). NAC, bovine serum albumin, Na-casein, sodium azide (NaN_3_), and Tween 20 were obtained from FUJIFILM Wako Pure Chemical Corporation (Osaka, Japan). A quadrivalent FLUBIK HA Syringes™ vaccine (The Research Foundation for Microbial Diseases of Osaka University, Suita, Japan), Poly(I:C) HMW vaccine grade (Invitrogen, Waltham, MA, USA), and an ELAST enzyme-linked immunosorbent assay (ELISA) amplification system (PerkinElmer, Inc., Waltham, MA, USA) were also used. Biotin-labeled (BT) monkey IgA (Mabtech, Inc., Cincinnati, OH, USA), biotin-labeled (BT) monkey IgA (alpha-chain) (Merck, Darmstadt, Germany), horseradish peroxidase (HRP)-human IgG (EY Laboratories, Inc., San Mateo, CA, USA), and BT IgE antibodies (Bio-Rad Laboratories, Inc., Hercules, CA, USA) were used.

Furthermore, the RNAiso Plus, PrimeScript™ Reverse Transcriptase, Recombinant RNase Inhibitor, TBGreen^®^ Premix Ex Taq™ (Tli RNaseH Plus), RR420 (Takara Bio Inc., Kyoto, Japan), RNeasy MinElute Cleanup Kit (QIAGEN, Tokyo, Japan), dNTP Mix and Oligo (dT)15 Primer (Promega, Tokyo, Japan), Low Input Quick Amp Labeling Kit, RNA6000 Nano Kit, Agilent Whole Human Genome DNA Microarray 4x44K v2, and Agilent Gene Expression Hybridization Kit (Agilent Technologies, Santa Clara, CA, USA) were used in this study.

### 2.2. Animals

This research consists of a total of nine cynomolgus macaques (*Macaca fascicularis*), consisting of both male and female individuals aged between 8 and 19 years. Adhering to the 3R policy (Replacement, Reduction, and Refinement) for animal usage, the macaque monkeys underwent a 20 month period of cleansing following the completion of specified exams. Prior to being utilized in the present study, these monkeys underwent testing for hepatitis B virus, Simian immunodeficiency virus, TB, *Shigella* spp., *Salmonella* spp., and helminthic parasites, all of which yielded negative results.

The animal exams were conducted in compliance with the rules established by the Institutional Animal Care and Committee Guide of Intelligence and Technology Lab, Inc. (ITL), which comply with the rules for Proper Conduct of Animal Experiments. The Animal Care Committee of ITL authorized these exams, assigning them the code AE2022022 and approving them on 24 November 2022. The ITL Biosafety Committee has also authorized more research.

### 2.3. Vaccination and Sampling

The vaccination antigen contained 120 μg /mL HA, i.e., 30 μg /mL of each HA subtype, including A/Victoria/1/2020 (H1N1), A/Darwin/9/2021 (H3N2), B/Phuket/3073/2013 (Yamagata), and B/Austria/1359417/2021 (Victoria). The HA antigen and Poly(I:C) adjuvant (1 mg/mL) were stored at 4 °C and −70 °C, respectively, until use. Nine cynomolgus macaques were separated into two groups. The first group, called the control/PBS group, consisted of four monkeys (animal ID numbers: pP01~03, qP09) who were sublingually administered PBS. The second group, called the experiment/HA + Poly(I:C) group, consisted of five monkeys (animal ID numbers: pP05–08, qP10) who were sublingually administered the HA + Poly(I:C) vaccination. The animals received either 0.5 mL of PBS for the control group or 0.5 mL containing 30 μg HA antigen and 400 μg Poly(I:C) for the experimental group.

**Figure 1:**
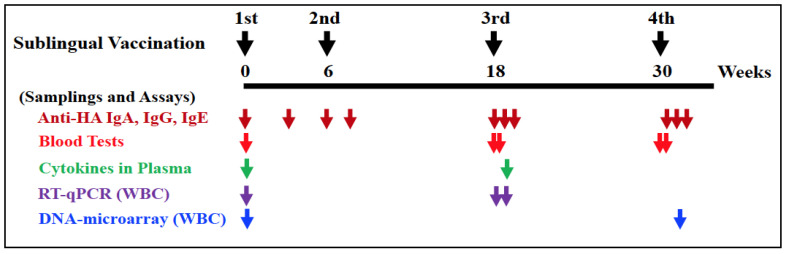
Points in time for sublingual vaccinations and assessments of anti-HA (hemagglutinin) antibodies, blood tests, plasma cytokines, quantitative reverse transcription PCR (RT-qPCR), and DNA microarray analyses. RT-qPCR and DNA microarray analyses were conducted with RNA isolated from white blood cells (WBC). Vaccinations were performed four times at 0 (1st), 6 (2nd), 18 (3rd), and 30 (4th) weeks. Arrows indicate sampling timepoints for each assay.

Vaccination procedures were conducted under anesthesia using a mixture of medetomidine and ketamine, with atipamezole administered to awaken the monkeys. Before vaccination, the sublingual surface of the monkeys was pretreated for 5 min using wet cotton dipped in 1.5% NAC, followed by saline wash to disintegrate the mucin layer. After the wet mucin was squeezed out using dry cotton, 0.5 mL of PBS or HA with Poly(I:C) was pipetted into the cavity under the tongue. After that, the mucin had to be left alone for a minimum of five minutes. Two sublingual vaccinations were performed: at 0 W and 6 W with a six-week interval, followed by the third and fourth vaccinations (at 18 W and 30 W) with twelve-week intervals, as shown in Figure 1.

As mentioned earlier, blood, saliva, and nasal washings were obtained from each anesthetized monkey. Plasma samples were generated by the centrifugation of blood in order to assay HA-specific IgA, IgG, and IgE antibodies. Saliva samples were absorbed onto a swab that contained polystyrene fiber, whereas nasal washings were obtained by centrifugating them in a centrifuge using a spin column. The samples were stored at a temperature of −40 °C until they were used.

### 2.4. ELISA for HA-Specific Antibodies

The ELISA to detect HA-specific IgA, IgG, or IgE antibodies was performed under the conditions mentioned in the previous report [8]. Briefly, the module plates were added with 100 mL of 0.4 mg/mL HA antigen with a 300-fold dilution of the vaccination antigen and incubated at 37 °C for 1 h, followed by incubation at 4 °C overnight.

After washing with PBS containing 0.05% Tween 20, the plates were added with 1% Na-Casein in PBS containing 0.02% NaN_3_ and then were incubated at 37 °C for 1 h, followed by storage at 4 °C until use. Samples (saliva, nasal washes, and plasma) obtained from both control/PBS and experiment/HA + Poly(I:C) groups at the time points shown in Figure 1 were diluted 100- to 500-fold with 1% Na-casein-PBS containing 0.02% NaN_3_. Fifty μL of the diluted samples and 1M NaCl at a final concentration of 0.5 M were added to the plates to eliminate non-specific reactions. PBS was also used as a negative control for the ELISA.

After incubation at 37 °C for 1 h or at 4 °C overnight and removing the samples, plates were washed with PBS containing 0.05% Tween 20. Subsequently, diluted BT monkey IgA, BT monkey IgA (alpha-chain), HRP-human IgG, or BT IgE antibodies were added for antibody detection and incubated at a temperature of 37 °C for 1 h. After washing, the plates were amplified with a diluted SA-HRP and the ELAST System containing biotinyl tyramide. This amplification resulted in a ten- to thirty-times enhancement of ELISA sensitivity. Following amplification, the plates were rinsed with PBS solution containing 0.05% Tween 20, and diluted SA-HRP was added, followed by incubation at 37 °C for 1 h. Color development was performed with TMB and terminated by adding H_2_SO_4_. Absorption was measured at 450 and 600 nm using a plate reader (iMark Microplate reader, Bio-Rad Laboratories, Inc., Hercules, USA).

The concentrations of total sIgA in saliva and nasal washings were measured using a Monkey IgA ELISA Basic Kit (Mabtech, Inc., Cincinnati, OH, USA) according to the manufacturer’s procedure.

A relative titer for HA-specific sIgA was estimated from a calculation of the optical density of HA-specific sIgA/concentrations of total sIgA, as the sIgA secreted in saliva and nasal washings varied from sample to sample.

### 2.5. Blood Testing

As previously reported [8], blood tests were performed. Blood samples from both groups at the time points shown in Figure 1 were taken one day before the first vaccine (W0), and one day before and after the third (W18) and fourth (W30) vaccinations. A complete blood count of eight items was performed on fresh whole blood samples: red blood cells, white blood cells (WBC), hemoglobin, hematocrit, mean cell volume, mean corpuscular hemoglobin, and platelets. Plasma samples were tested for thirteen biochemical blood tests, including total protein, albumin, albumin/globulin ratio, total bilirubin, aspartate and alanine transaminases, alkaline phosphatase, gamma-glutamyl transpeptidase, urea nitrogen, creatinine, total cholesterol, neutral fats, and C-reactive protein.

### 2.6. Cytokine Testing

The level of plasma cytokines was assessed using plasma samples obtained from the experiment/HA + Poly(I:C) group at the time points shown in Figure 1, one day before to the initial immunization (W0: pre-vaccination) and one week after the third vaccine (W19: post-vaccination). The levels of three cytokines, namely IFN-alpha, IFN-gamma, and IL-17, were determined using ELISA. The Monkey IFN-alpha (pan) ELISA Kit (Arigo Biolaboratories, Zhubei, Taiwan), the Monkey IFN-gamma ELISA Kit and Monkey IL-17 ELISA Kit (U-CyTech Biosciences, Utrecht, The Netherlands) were used, respectively. The control sample in each assay kit was used for negative control of this cytokine assay.

### 2.7. Isolation of WBCs

Blood was collected using heparinized syringes one day before the first vaccination (W0), prior to (W18) and 1 week after (W19) the third, and 1 week after the fourth (W31) vaccinations, as shown in Figure 1. White blood cell (WBC) samples were prepared by using erythrocyte lysis solution, following the method reported by Hoffman et al. [12].

### 2.8. Isolation of RNAs

In order to isolate total RNA, WBCs were homogenized with RNAiso Plus (Takara Bio Inc., Kyoto, Japan), and total RNA was extracted, as described in previous paper [13]. Then, RNA was treated with DNase using QIAGEN’s reagent in the aqueous phase, and was subsequently purified using the RNeasy MinElute Cleanup Kit from QIAGEN, following the instructions of the manufacturer. The quantity of this obtained RNA was assessed spectrophotometrically at wavelengths of 230, 260, 280, and 320 nm using an Ultrospec 2000 spectrometer (GE Healthcare Biosciences AB, Uppsala, Sweden).

The RNA integrity number (RIN) for DNA microarrays was determined using an Agilent 2100 Bioanalyzer (Agilent Technologies Japan Ltd.). The microarray examination only utilized RNA samples of exceptional quality, with an A260/A230 ratio of 1.5, an A260/A280 ratio of 1.8, and a RIN value of 6.0.

### 2.9. Quantitative Reverse Transcription PCR for Gene Expression Analyses

The mRNA levels of the target genes in WBC samples were quantified using quantitative reverse transcription PCR (RT-qPCR), as described in a previous paper [14]. Simply, complementary DNA (cDNA) was made from the purified RNA using PrimeScript Reverse Transcriptase with RNase Inhibitor (Takara Bio Inc.), dNTPs mixture (Promega Corp.), and Oligo dT primers (Invitrogen). The Mx3000P QPCR System (Agilent Technologies Inc) was used for real-time PCR analysis. The Takara Bio Inc. SYBR Premix Ex Taq II (Tli RNaseH Plus) Kit was utilized in conjunction with the system. Primer sequences for monkey IL12a, IL12b, GZMB, type I IFNs (IFN-alpha1 and IFN-beta1), CD69, and the reference gene low-density lipoprotein receptor-related protein 10 (Lrp10) were designed with Primer3 and Primer-BLAST [14]. To determine the cDNA copy number of the genes, a standard curve was generated by diluting a known quantity of glyceraldehyde 3-phosphate dehydrogenase amplicon in a series. The PCR protocol involved an initial denaturation step at 95 °C for 15 s, followed by 35 cycles of denaturation at 95 °C for 10 s and annealing/extension at 63 °C for 30 s. A dissociation curve was also included. The quantification of target gene mRNA was determined by comparing it to the expression of an appropriate reference gene, LRP10 [15].

### 2.10. DNA Microarray for High-Throughput Gene Expression Analyses

A DNA microarray analysis was conducted on both the control and experimental groups. Following the synthesis of cDNA, Cy3-labeled complementary RNA (cRNA) was generated and purified using the Low Input Quick Amp Labeling Kit (Agilent Technologies, Santa Clara, CA, USA) following the instructions from the manufacturer. Specifically, reverse transcription was conducted using a T7 promoter-oligo(dT) primer. The absorbance at wavelengths of 260, 280, 320, and 550 nm was measured to verify that the labeled cRNA had a concentration of more than 6 pmol/mg of Cy3-CTP. The labeled cRNA was subsequently fragmented using the Gene Expression Hybridization Kit (Agilent Technologies, Santa Clara, CA, USA) and then set onto Whole Human Genome DNA Microarray 4× 44K v2 slides (Agilent Technologies, Santa Clara, CA, USA). After hybridization at 65 °C for 17 h, the slides were cleansed using Gene Expression Wash Buffers 1 and 2 (Agilent Technologies) according to the instructions provided by the manufacturer. The slides were subsequently scanned using a GenePix 4000B scanner (Molecular Devices, San Jose, CA, USA). The software GenePix Pro 6.0 (Molecular Devices) was utilized to convert scanned pictures into digital format and standardize them.

### 2.11. Bioinformatic for Microarray Data Analyses

Genes that showed a greater than 2-fold upregulation or less than a 0.5-fold downregulation in expression compared to the control group were identified in the experimental group. These genes were annotated, and Google, NCBI databases, and other relevant information sources were utilized to search for references. We deduced the immune-related functions of the annotated genes to understand the potential mechanisms of action for the sublingual vaccine.

### 2.12. Data Expression and Statistical Analysis

Data were expressed as a mean standard deviation (SD). Comparisons were made using a Student’s *t*-test if the variance was equal, and Welch’s *t*-test if the variance was unequal.

## 3. Results

### 3.1. Complete Blood Count, Biochemical Blood Test, and Plasma CRP

Blood or plasma samples from the control/PBS and experiment/HA + Poly (I:C) groups underwent blood tests one day before the first vaccination (W0) and one day before and after the third (W18) and fourth (W30) vaccinations (Figure 1). In the HA + Poly (I:C) group, there was a slight increase in the levels of the eight items in the complete blood count and the thirteen items in the biochemical blood test one day after vaccination.Similarly, the control/PBS group also showed a mild increase in these levels. The similarities between the experiment and control groups remained consistent at all time points. Furthermore, plasma CRP levels in the experiment/HA + Poly (I:C) group increased 2.5-fold one day after vaccination, while those in the control/PBS group also increased 3-fold. These indicated that the increase in CRP was most likely due to experimental stress rather than the vaccine itself.

These results suggest that sublingual vaccinations using the Poly(I:C) adjuvant is safe, as it does not cause any notable adverse effects in nonhuman primates.

### 3.2. HA-Specific Antibodies

**Figure 2:**
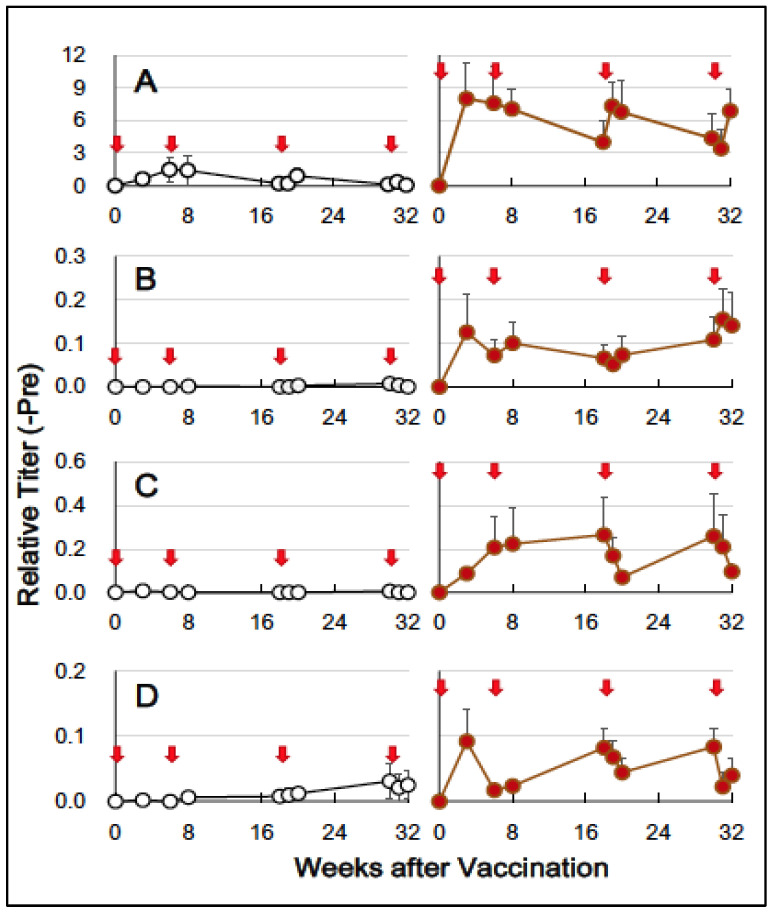
HA-specific antibodies induced using the sublingual vaccination with HA + Poly(I:C). (**A**) Anti-HA sIgA in saliva, (**B**) Anti-HA sIgA in nasal washings, (**C**) Anti-HA IgA in plasma, and (**D**) Anti-HA IgG in plasma. Open circles (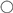) indicate the control group, and solid brown circles (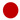) indicate the experiment/HA + Poly(I:C)) group. Red arrows (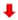) indicate vaccination. Relative titers were estimated using the conditions mentioned in Section 2.

We collected saliva, nasal washings, and plasma ten times during the course of this study, and assayed the relative titers of anti-HA-specific sIgA in saliva and nasal washings and anti-HA-specific IgA and IgG in plasma. After the first vaccination, the HA + Poly (I:C) group showed a significant level of anti-HA sIgA in their saliva (Figure 2A). We also detected anti-HA sIgA in nasal washings (Figure 2B). A low level of anti-HA IgA and IgG was seen in plasma of the HA + Poly (I:C) group after the first vaccination (Figure 2C,D). In anti-HA sIgAs of saliva and nasal washings, apparent booster effects and waning of the elicited anti-HA sIgAs were not observed after the first vaccination until the end of the fourth vaccination (Figure 2A,B). Anti-HA IgE in plasma was undetectable at any time point.

The levels of anti-HA s-IgA in saliva and nasal washings, as well as anti-HA IgA and IgG in plasma, appeared to be unchanged at any time point of the vaccinations in the control group, as shown in Figure 2A–D.

These findings indicate that the sublingual vaccine containing the Poly(I:C) adjuvant triggered an immune response, resulting in the production of antigen-specific antibodies in saliva, nasal cavities, and blood. The sublingual vaccine appears to be relatively effective at the vaccination site (oral cavity) compared to a distinct site (nasal cavities).

### 3.3. Production of Inflammation-Associating Cytokines

**Figure 3:**
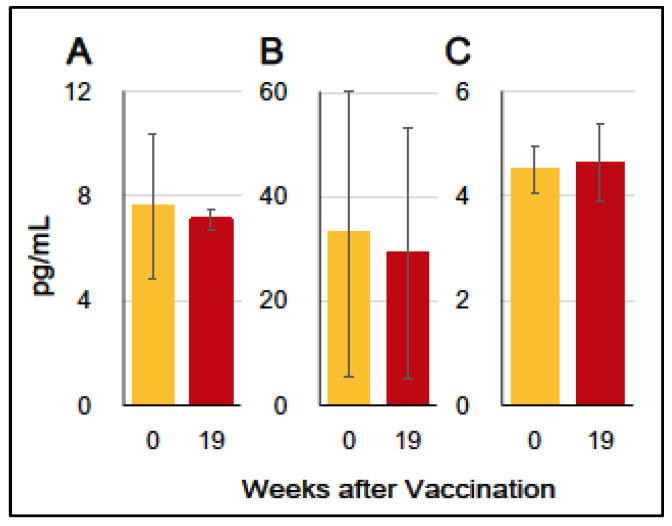
Cytokine production in the experiment (HA + Poly(I:C)) group at the commencement of vaccination (W0:0), and 1 week after the third vaccination (W19:19). (**A**) IFN-alpha, (**B**) IFN-gamma, and (**C**) IL-17. The levels of cytokines were expressed as pg/mL of plasma.

To assess immune-proinflammatory responses in the experiment (HA + Poly(I:C)) group, we examined three inflammation-associated cytokines, (IFN-alpha, IFN-gamma, and IL-17) produced in blood at one day before the first vaccination (W0) and one week after the third vaccination (W19). As shown in Figure 3A–C, the levels of IFN-alpha, IFN-gamma, and IL-17 in plasma at W19 (post-vaccination) did not increase compared to those at W0 (pre-vaccination), suggesting that the sublingual vaccination scarcely affected the production of these cytokines in the blood.

### 3.4. Gene Expression of Immuno-Proinflammatory Factors

**Figure 4:**
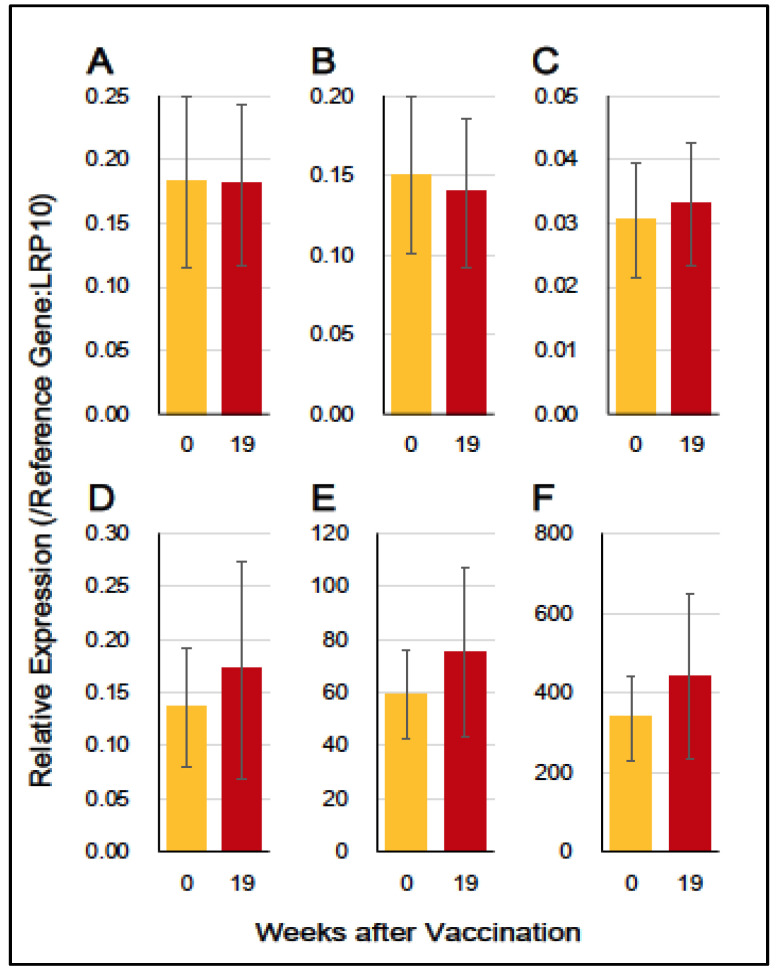
Gene expression (mRNA) levels of cytokines and effector molecules in the experiment (HA + Poly(I:C)) group at the time points: pre-vaccination (W0:0), and 1 week after (W19:19) the third vaccination. (**A**) *IL12A*, (**B**) *IL12B*, (**C**) *IFNA1*, (**D**) *IFNB1*, (**E**) *CD69*, and (**F**) *GZMB*. Red arrows indicate vaccination.

We assessed the gene expression of six immuno-proinflammatory factors (*IL12A*, *IL12B*, *IFNA1*, *IFNB1*, *CD69*, and *GZMB* genes) in the experiment (HA + Poly(I:C)) group. These gene expression analyses were performed by means of RT-qPCR using RNA samples from WBCs at three time points: pre-vaccination (W0), and a week after (W19) the third vaccination. As shown in Figure 4, the expression levels of all six genes (*IL12A*, *IL12B*, *IFNA1*, *IFNB1*, *CD69*, and *GZMB*) appeared to be neither upregulated nor downregulated at W19 in response to the third vaccination at W18.

These findings suggest that the sublingual vaccination with HA antigen and the Poly(I:C) adjuvant has little effect on the gene expression of these immuno-proinflammatory factors in WBCs.

### 3.5. Microarray Analyses and Bioinformatics

We aimed to elucidate the immunological mechanisms underlying the response elicited by the sublingual vaccination with the Poly(I:C) adjuvant. We performed DNA microarray analyses using WBC samples collected at pre-vaccination (W0) and one week after the fourth vaccination (W31) for both the control and experiment groups (Figure 1). Table 1 and Table 2 present the genes associated with immune-related responses that were upregulated more than two-fold or downregulated by less than half, respectively, in the experiment group compared to the control group.

#### 3.5.1. Upregulated Genes

Five genes, namely *CLEC4G*, *PF4V1*, *KLF1*, *OLFM1*, and *GNG11*, were significantly upregulated in response to the sublingual vaccination (Table 1). *CLEC4G* encodes a calcium-dependent glycan-binding protein (C-type lectin) expressed on the surface of immune cells. The product of *PF4V1* is also known as C-X-C motif chemokine 4 ligand 1 (CXCL4L1). The upregulation of *CLEC4G* and *PF4V1* is understood to have enhanced the immunological responses observed in this study. *KLF1* encodes a transcription factor that transcribes the beta-globin gene in erythroid cells. *OLFM1* is abundantly expressed in the nervous system. The immunological relevance of *KLF1* and *OLFM1* is not as evident as that of *CLEC4G* and *PF4V1*. *GNG11* encodes a member of the guanine nucleotide-binding protein (G protein) gamma family, and the upregulation of *GNG11* may have suppressed the immune response against the vaccinated antigen.

#### 3.5.2. Downregulated Genes

Six genes, namely *NEURL1B*, *CHST15*, *MOB3A*, *ANXA6*, *DNAJA3*, and *HSPH1*, were significantly downregulated in response to the sublingual vaccination (Table 2). *NEURL1B* encodes a ubiquitin protein ligase, and is expressed in the nervous system. Based on our previous results [8], the downregulation of *NEURL1B* was implicated in the observed immunological responses. CHST15 transfers a sulfate group to position six of *N*-acetylgalactosamine 4-sulfate in chondroitin sulfate and dermatan sulfate, and *CHST15* is expressed in B-cell lineage. *ANXA6* is abundantly expressed in T-cells, and ANXA6 is an important component of T-cell plasma membrane. The downregulation of *CHST15* and *ANXA6* suppressed the immunological responses observed in this study. *MOB3A* encodes a protein kinase activator. *DNAJA3* and *HSPH1* encode heat shock proteins (HSPs). As discussed below, *MOB3A*, *DNAJA3*, and *HSPH1* appear to affect the immune response in this study.

## 4. Discussion

### 4.1. Poly(I:C) Adjuvant

The sublingual vaccine using the influenza HA antigen and the Poly(I:C) adjuvant demonstrated safety in nonhuman primates, as evidenced by the results of blood tests, including chemical tests, in this study. These findings align with our previous research, which indicated the safety of the sublingual vaccine containing the SARS-CoV-2 S RBD peptide antigen and the same adjuvant [8]. Despite Poly(I:C) being utilized as a vaccine adjuvant in cancer treatment, its clinical use remains unapproved beyond limited applications. Safety concerns regarding Poly(I:C) adjuvants have primarily arisen from studies involving nasal vaccination in mice [31,32]. Notably, humans differ anatomically from mice in lacking defined bronchi-associated lymphoid tissue (BALT), but possessing infection-inducible BALT [33]. Moreover, differences in the immune systems between rodents and primates have been underscored by genome-based evidence [34]. Hence, adverse events mediated by Poly(I:C) may be overestimated when administered nasally in rodent models. Research into the development of Poly(I:C) as a vaccine adjuvant continues in both preclinical and clinical settings [35].

As a danger signal, double-stranded Poly(I:C) RNA activates APCs, particularly dendritic cells (DCs) [36,37]. Poly(I:C) binds to endosomal TLR3 and cytosolic receptors, such as retinoic acid-inducible gene I and melanoma differentiation-associated gene 5 [38,39]. Activation of TLR3 by Poly(I:C) leads to the production of proinflammatory cytokines, IFNs, IL-15, and Natural killer (NK) cell activation [40]. Previous studies primarily reported the Poly(I:C)-mediated production of proinflammatory cytokines and related factors in studies involving nasal vaccination in mice [31,32]. However, when employing sublingual vaccination in nonhuman primates, we observed minimal upregulation in the gene expression of proinflammatory cytokines and related factors, including *IL12A*, *IL12B*, *IFNA1*, *IFNB1*, *CD69*, and *GZMB* (Figure 4).

### 4.2. Sublingual Poly(I:C)-Adjuvanted Vaccination

Unlike the skin, the sublingual epithelium in humans and monkeys lacks keratinized cell layers, allowing the antigens to penetrate the mucosa without specialized devices like microneedles [41]. Antigens are captured by antigen-presenting DCs, primarily Langerhans cells, dispersed in the mucosa. For example, the ovalbumin antigen was captured by sublingual DCs within 30–60 min after sublingual administration in mice [42]. Studies by Hervouet et al. have demonstrated the presence of antigen-bearing DCs in distant lymph nodes and the spleen [43]. Typically, humoral immune responses can be observed two weeks after sublingual immunization in mice [44]. In this study, anti-HA-specific IgA antibodies were detected in saliva and nasal washings three weeks after sublingual vaccination of cynomolgus monkeys (Figure 2B,C). Anti-HA IgG antibodies were also detected in the blood (Figure 2D). These results suggest that sublingually administered HA antigen formulated with the Poly(I:C) adjuvant could elicit mucosal immune responses at remote sites, leading to a systemic immune reaction. In our previous study, IgA and IgG antibodies against the SARS-CoV-2 spike RBD were raised in the blood after sublingual vaccination in a non-human primate model [8].

### 4.3. Genes Upregulated by Sublingual Vaccine with HA Antigen and Poly(I:C) Adjuvant

An efficient method for studying the expression levels of most genes in an organism’s genome is DNA microarray studies, which are a kind of high-throughput research. Using the DNA microarray approach, we were able to deduce molecular processes in the immune system and their responses. These events were triggered by a sublingual vaccination that contained two strains of influenza A and B viruses’ HA antigens and the Poly(I:C) adjuvant. The DNA microarray analyses were performed using RNA extracted from WBCs, since WBCs are easily available for future clinical studies and can be compared with current preclinical studies conducted on nonhuman primates. The results showed that in the vaccinated group, five genes (*CLEC4G*, *PF4V1*, *KLF1*, *OLFM1*, and *GNG11*) were significantly upregulated (more than two-fold) compared to the control group (Table 1).

*CLEC4G* encodes a C-type lectin that binds to complex-type *N*-glycans. In humans, *CLEC4G* is located on chromosome 19 and is clustered with three genes encoding DC-SIGN, L-SIGN, and CD23, all of which are C-type lectins. CLEC4G serves as an attachment site for the Ebola filovirus and West Nile flavivirus, and it also acts as a receptor for PAMPs of SARS-CoV-2 [16]. Regarding influenza virus, DC-SIGN serves as an entry site for influenza A virus into DCs [45]. Recently, Lu et al. demonstrated that myeloid cells, including monocytes, macrophages, and DCs, express *CLEC4G*, and its binding to ligands activates inflammatory reactions in these cells [17]. Notably, we previously sublingually vaccinated cynomolgus macaques with the RBD peptide of SARS-CoV-2 S glycoprotein, and found that *CLEC4G* was significantly downregulated in WBC [8]. However, Lu et al. reported that CLEC4G does not bind to the RBD region of the SARS-CoV-2 S protein. This discrepancy between the upregulation of CLEC4G by the influenza HA antigen and its downregulation by the SARS-CoV-2 S RBD peptide antigen warrant further investigation.

Previously, *CLEC4G* expression was described as restricted to sinusoidal endothelial cells of the liver and lymph nodes. Tang et al. revealed that CLEC4G is a novel T-cell regulator suppressing the effector functions of activated hepatic T cells [46]. The liver favors the induction of tolerance rather than immunity, which is critical for maintaining immunologic silence in response to harmless antigenic material present in food. The sublingual epithelium is considered to be an immunological tolerance-prone site, and sublingual application of allergens is a curative therapy for allergic disorders and diseases [47]. As this study did not collect and examine the sublingual tissue samples, it is uncertain whether *CLEC4G* is sublingually expressed and how its expression levels change at the site of administration after sublingual immunization.

After in vitro stimulation with GM-CSF and IL-4, *CLEC4G* is upregulated in monocyte-derived macrophages and DCs, and a splice variant encoding a soluble form of CLEC4G, lacking the transmembrane region, is preferentially synthesized [18]. As mentioned above, *CLEC4G* is clustered with the CD23-encoding gene (*FCER2*). CD23, a C-type lectin, is also known as a low-affinity IgΕ receptor (FcεRII) on B cells. Splice variants of CD23 are translated into soluble and membrane-bound forms. Soluble CD23 binds to CD21 on B cells and stimulates IgE production, while membrane-bound CD23 binds to the IgE ligand and suppresses IgE production [48]. Like CD23, soluble and membrane-bound CLEC4G may exert contradictory influences on immune reactions.

PF4V1 is contained in platelets and well-known as a potent inhibitor of angiogenesis. PF4V1 (CXCL4L1) is a ligand of the CXCR3 receptor and chemoattracts activated T cells, NK cells, and immature DCs [19]. Recently, we found the upregulation of *PF4V1* after sublingual vaccination using the SARS-CoV-2 S RBD peptide antigen formulated with the Poly(I:C) adjuvant [8]. The upregulation of *PF4V1* observed in the present study using the influenza HA antigen and the same adjuvant suggests that the *PF4V1* induction is not antigen-specific, but is related to the inflammatory effect of the Poly(I:C) adjuvant. Brandhofer et al. reported that PF4V1 forms a heterocomplex with an atypical chemokine macrophage migration inhibitory factor (MIF), and also that the PF4V1-MIF complex does not have chemotactic activity to T cells and monocytes [49]. The upregulation of PF4V1 may have a suppressive aspect for inflammation caused by adjuvants.

KLF1 is a zinc-finger transcription factor that is indispensable to the transcription of the β-globin gene in hematopoietic cells. *Klf1*, the murine ortholog of *KLF1*, upregulates *Cd274*, which encodes programmed death ligand 1 (PD-L1) in regulatory T cells (Tregs) [20]. In this study, the upregulation of *KLF1* in WBC may have contributed to Treg induction and immunological tolerance. However, we did not observe the upregulation of either *CD274* or *PDCD1* encoding the PD-1 receptor, whose ligand is PD-L1.

In colorectal cancer (CRC) cells, OLFM1 inhibits the non-canonical nuclear factor-kappa B (NF-κB) signaling pathway, which plays a pivotal role in the proliferation and activation of immune cells [21]. *GNG11* is downregulated in splenic marginal zone lymphomas [22], suggesting that upregulated *GNG11* has an inhibitory effect on the proliferation of B cells. The upregulation of *OLFM1* and *GNG11* in WBC observed in the present study may have suppressed the immune response against the vaccinated antigen.

### 4.4. Genes Downregulated by Sublingual Vaccine with HA Antigen and Poly(I:C) Adjuvant

The results of the DNA microarray analyses using WBC RNA showed that in the vaccinated group, six genes, namely *NEURL1B*, *CHST15*, *MOB3A*, *ANXA6*, *DNAJA3*, and *HSPH1*, were significantly downregulated (over 1/2-fold) compared to the control group (Table 2).

NEURL1B ubiquitinates Delta, a ligand of Notch in the Notch signaling pathway, and accelerates its proteasomal degradation. NEURL1B plays a pivotal role in the embryonic development of the nervous system, and mRNA levels of *NEURL1B* subside postnatally [23]. *NEURL1B* is downregulated in CRC tissues, and this downregulation is implicated in the proliferation, invasion, and metastasis of CRC cells [24]. Recently, we observed the downregulation of *NEURL1B* after sublingual vaccination with the SARS-CoV-2 S RBD peptide and the Poly(I:C) adjuvant [8]. The downregulation of *NEURL1B* in this study using the HA antigen and the same adjuvant suggests that the observed downregulation is caused by the Poly(I:C) adjuvant. Similar to CRC cells, the downregulation of *NEURL1B* may be involved in the proliferation of, secretion of proteases from, and motility of inflammatory cells.

*CHST15* is expressed in pre-B cells, pro-B cells, and mature B cells in the B cell lineage, and upregulates the recombination-activating gene-1 [25]. The downregulation of *CHST15* observed in this study may have interfered with the humoral immune response against the HA antigen.

*MOB3A* encodes a protein kinase activator, and is involved in intracellular signal transduction. In normal cells, aberrant production/activation of RAS, BRAF, or MEK leads to an irreversible cell-cycle arrest termed oncogene-induced senescence (OIS). MOB3A bypasses OIS by engaging the Hippo pathway, which plays a central role in the regulation of the size of various organs [50]. Macrophage stimulating 1 (MST1), a member of the Hippo pathway, inhibits the differentiation of follicular helper T cells and the germinal center (GC) reaction [26]. Moreover, the downregulation of *MOB3A* may have stimulated immune response through GC formation. However, *MST1* was not significantly downregulated in this study.

ANXA6 is implicated in the proliferation of and intracellular signaling in T cells. In vivo proliferation of CD4+ T cells, but not CD8+ T cells, was impaired in *AnxA6*^−/−^ mice [27]. Our findings suggest that the downregulation of *ANXA6* may disturb helper T-cell function and humoral immune responses.

DNAJA3, an HSP localized to mitochondria, stimulates the ATPase activity of Hsp70 chaperones and facilitates protein folding, degradation, multimeric complex assembly, and so on. Activation of NF-κB, a pivotal transcription factor in immune cells, was suppressed by *DNAJA3* knockdown in vitro [28]. HSPH1 promotes the replacement of Hsp70-bound ADP with ATP and facilitates the chaperone activity of HSP70. Additionally, HSPH1 plays a distinct role as a holdase that inhibits the aggregation of misfolded proteins and as a disaggregase that resolubilizes aggregates for new folding attempts or proteasomal degradation [29]. HSPH1 stimulates NF-κB signaling through MyD88 stabilization in activated B cell diffuse large B cell lymphoma [30]. Both DNAJA3 and HSPH1 activate the NF-κB signaling pathway in certain circumstances. The downregulation of *DNAJA3* and *HSPH1* observed in this study may have a negative effect on the immune response after vaccination.

### 4.5. A Balance State of Immune-Enhancing and -Suppressive Response Induced by the Sublingual Vaccine

**Figure 5:**
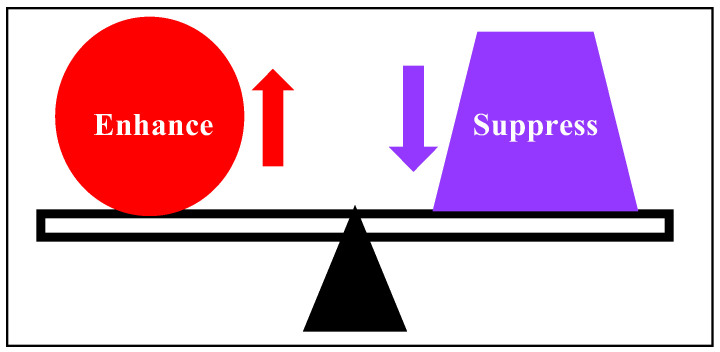
Balanced immune response elicited by the sublingual vaccine with an HA antigen and Poly(I:C) adjuvant.

Therefore, the regulations of *CLEC4G* and the functions of CLEC4G in sublingual vaccination of non-human primates are worth further investigation. The gene expression profile lacked a clear direction as a whole; that is, both potentially immune-enhancing and immune-suppressive changes in mRNA composition were observed in response to the sublingual administration of the HA antigen and the Poly(I:C) adjuvant. Potentially, the inflammatory reaction was enhanced by the upregulation of *CLEC4G*, and the acquired immune reaction was inhibited by the downregulation of *ANXA6*. The NF-κB signaling pathway seems to be inhibited by the upregulation of *OLFM1* and the downregulation of *DNAJA3* and *HSPH1*. These observations suggest that the sublingual vaccine yields a balanced state of immune-enhancing and immune-suppressive responses, as seen in a previous study (8) (Figure 5).

## 5. Conclusions

The sublingual vaccine, comprising the influenza HA antigen and the Poly(I:C) adjuvant, demonstrated safety in a nonhuman primate model. This vaccine effectively stimulated the production of HA-specific sIgA antibodies in saliva and nasal washings, while also eliciting the production of anti-HA IgG and IgA antibodies in the blood. Notably, the sublingual vaccine had minimal impact on the gene expression of proinflammatory cytokines and related factors, as assessed using an RT-qPCR of WBC. Additionally, DNA microarray analyses suggested a complex interplay of both immune-enhancing and immune-suppressive changes in the gene expression profile induced by sublingual vaccination in cynomolgus monkeys.

## Figures and Tables

**Table 1:** The genes exhibited an upregulation of more than two-fold using the sublingual vaccination with HA+Poly (I:C).

Gene Symbol	Fold Change	Product. Description. Function [Reference].	Expected Effect *
*CLEC4G*	2.2	C-type lectin. Receptor of PAMP [16]. Activation of inflammatory reactions [17]. Transcript variants [18].	**↑**
*PF4V1*	2.2	Also known as CXCL4L1. Chemoattractant of T and NK cells [19].	**↑**
*KLF1*	2.1	Zn-finger transcription factor. Transcription of γ-globin gene. Upregulation of *CD274* in Tregs [20].	**↓**
*OLFM1*	2.1	Nervous system. Inhibition of non-canonical NF-κB pathway in CRC [21].	**↓**
*GNG11*	2.0	G protein gamma family. Downregulated in splenic marginal zone lymphomas [22].	**↓**

The upregulation fold was estimated using relative gene expression calculated from that of pre-vaccination (W0) to post-7-days after the fourth vaccination (W31). * Expected effect indicates whether the gene expression change, either upregulation or downregulation, is expected to enhance (**↑**) or suppress (**↓**) the immune response and its related responses. Description, function, and reference(s) of these genes are explained in brown color.

**Table 2:** The genes exhibited an upregulation of more than two-fold with the sublingual vaccination with HA+Poly (I:C).

Gene Symbol	Fold Change	Product. Description. Function [Reference].	Expected Effect *
*NEURL1B*	0.45	Ubiquitin protein ligase. Development of the nervous system [23]. Downregulated in CRC tissues [24]. Downregulated by Poly(I:C) adjuvant [8].	**↑**
*CHST15*	0.45	Sulfotransferase. Expressed in B-cell lineage. Upregulation of RAG1 [25].	**↓**
*MOB3A*	0.48	Protein kinase activator. Inhibition of GC through the Hippo pathway [26].	**↑**
*ANXA6*	0.48	Expressed in T cells. Component of T-cell plasma membrane. Stimulation of helper T cells [27].	**↓**
*DNAJA3*	0.48	Mitochondrial HSP. Stimulation of ATPase activity of Hsp70. Activation of NF-κB [28].	**↓**
*HSPH1*	0.49	HSP. Replacement of Hsp70-bound ADP with ATP. Holdase and disaggregase activity [29]. Stimulation of NF-κB signaling in activated B-cell diffuse large B-cell lymphoma [30].	**↓**

The downregulation fold was estimated by relative gene expression calculated from that of prevaccination (W0) and post-7-days (W31) of the fourth vaccination. * Expected effect means that the gene expression change, either upregulation or downregulation, is expected to enhance (**↑**) or suppress (**↓**) the immune response and its related responses. Description, function, and reference(s) of these genes are explained in brown color.

## Data Availability

Data are available from S.N. upon reasonable request.
